# Emergent multicellular life cycles in filamentous bacteria owing to density-dependent population dynamics

**DOI:** 10.1098/rsif.2011.0102

**Published:** 2011-05-18

**Authors:** Valentina Rossetti, Manuela Filippini, Miroslav Svercel, A. D. Barbour, Homayoun C. Bagheri

**Affiliations:** 1 Institute of Evolutionary Biology and Environmental Studies, University of Zurich, Zurich, Switzerland; 2 Institute of Mathematics, University of Zurich, Zurich, Switzerland

**Keywords:** generation time, life cycle, evolution of multicellularity, evolution of development

## Abstract

Filamentous bacteria are the oldest and simplest known multicellular life forms. By using computer simulations and experiments that address cell division in a filamentous context, we investigate some of the ecological factors that can lead to the emergence of a multicellular life cycle in filamentous life forms. The model predicts that if cell division and death rates are dependent on the density of cells in a population, a predictable cycle between short and long filament lengths is produced. During exponential growth, there will be a predominance of multicellular filaments, while at carrying capacity, the population converges to a predominance of short filaments and single cells. Model predictions are experimentally tested and confirmed in cultures of heterotrophic and phototrophic bacterial species. Furthermore, by developing a formulation of generation time in bacterial populations, it is shown that changes in generation time can alter length distributions. The theory predicts that given the same population growth curve and fitness, species with longer generation times have longer filaments during comparable population growth phases. Characterization of the environmental dependence of morphological properties such as length, and the number of cells per filament, helps in understanding the pre-existing conditions for the evolution of developmental cycles in simple multicellular organisms. Moreover, the theoretical prediction that strains with the same fitness can exhibit different lengths at comparable growth phases has important implications. It demonstrates that differences in fitness attributed to morphology are not the sole explanation for the evolution of life cycles dominated by multicellularity.

## 1. Introduction

Multicellularity is an organizational characteristic present in the majority of organisms whose size surpasses microbial scales. Phylogenetic inference suggests that evolutionary transitions to multicellularity have occurred several times during the history of life [1–6]. Nonetheless, despite its fundamental importance, it is difficult to empirically study the evolutionary and ecological forces that may lead to a transition from single-celled to multicellular organization. One set of theoretical explanations are based on examining the consequences of a shift between units of selection. These approaches consider the changing boundaries of an individual after a transition to multicellularity, and how the different activities of component cells can potentially lead to synergies that increase the fitness of the multicellular assemblage [1,5,7,8]. Experimental results concerning social evolution in microbes are also largely compatible with this perspective [9–14].

An additional set of explanations for multicellularity are based on the potential selective advantages associated with increased size. Among the proposed size-related advantages are increases in the efficiencies of feeding [15–21], improved dispersal [16,22,23] and predator avoidance [24–28]. Although many of the proposed explanations are highly plausible, there is a lack of empirical validation. Some notable exceptions are measurements of motility and feeding efficiencies in Volvocalean green algae [20,22] and density-dependent growth in *Myxoccoccus xanthus* [17,21,29].

Multicellular bacteria with a filamentous form are ubiquitous in many environments, including freshwater, oceans, soil, extreme habitats and the human body [30–33]. Extensive empirical work has been done to examine and monitor filamentous bacteria that can be toxic or problematic in the environment [34–37]. Some filament-forming cyanobacteria also develop specialized terminally differentiated cells, named heterocysts, that fix nitrogen and allow for the division of labour [38,39]. Although differentiation can represent a clear evolutionary advantage, theoretical and phylogenetic evidence suggests that in the cyanobacterial case, undifferentiated multicellularity evolved prior to differentiated multicellularity [40]. In aquatic bacteria, the most common hypothesis for the advantage of filamentation—and hence undifferentiated multicellularity—is an increase in size and the concomitant defence against predation by grazers [25–27,41–43]. However, experiments indicate that the avoidance of predatory grazers is not the only factor causing an increased frequency of filamentous bacteria in aquatic environments [44]. Notably, in some bacterial species, filament formation appears to be dependent on the growth state of the population, whereby an increase in the dilution rate of chemostat cultures leads to longer filament lengths [27,45].

No theoretical studies address the distribution of filament lengths and the population dynamics leading to shorter or longer filaments, although differences in length can reflect the extent to which a species is able to maintain multicellularity. Environmental conditions such as temperature, solar irradiation and nutrient concentrations have been detected as factors in determining the mean size (filament length) of different cyanobacterial species [46–48]. Several of these factors also contribute to competition between species and adaptation to different niches [49,50]. Filament breakage can occur because of external mechanical stress, lytic processes initiated by pathogens [51–53], or programmed cell death [54–58].

**Figure 1.**
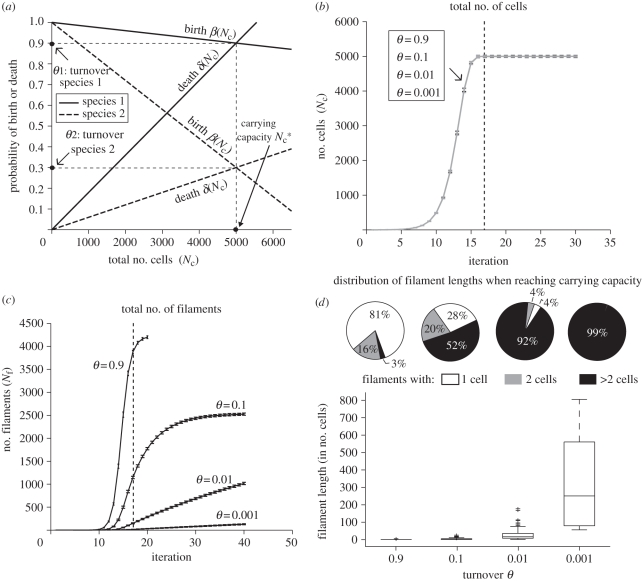
Simulation model (*a*) and results based on 1000 runs (*b*–*d*). (*a*) Schematic of birth and death rate functions dependent on total number of cells *N*_c_. The value of the population at which the two rates are the same is defined as the carrying capacity *N*_c_^*^. We refer to the value of the rates at the carrying capacity as the turnover rate *θ*. The plot illustrates that the same carrying capacity can be achieved by species that have a different turnover (solid lines, species 1; dashed lines, species 2). (*b*) For all birth and death functions following the scheme in (*a*) *N*_c_ reaches the steady state following the same growth curve despite the differing turnovers. The dashed line indicates the time at which the carrying capacity is reached (see mathematical definition of *N*_c_^*^ in §3.1.1). Error bars represent standard deviation of the mean. (*c*) When the population size reaches the carrying capacity (dashed line), the higher the turnover, the higher the total number of filaments *N*_f_. Nonetheless, after many more iterations, *N*_f_ reaches a steady state of about 2500 filaments for all sufficiently small turnovers (plot not shown). Error bars represent standard deviation of number of filaments. (*d*) Box plots of filament lengths when *N*_c_ reaches the carrying capacity. Populations with low turnover have higher median length values than populations with high turnover. The corresponding pie charts on the top show the proportion of single-celled, double-celled and longer filaments in the population.

In the present study, we deliberately avoid a modelling framework in which one assumes an *a priori* fitness advantage to multicellularity. We also do not aim to provide mechanistic explanations for the ‘origin’ of multicellularity. Rather, by considering cellular growth and division in a filamentous context, we investigate the constraints that would affect filament formation and the maintenance of multicellularity. This gives an indication of the capability of bacterial organisms to evolve multicellular life cycles according to their life-history traits. Our model is built on the basic assumption that the growth of a population of filamentous bacteria and the changes in the length of the filaments can be set in an ecological framework. In classical population dynamics, the change in population size is governed by the processes of birth and death (and sometimes migration), combined by the well-known logistic equation owing to Verhulst. In this model, birth and death rates are usually assumed to be decreasing and increasing functions of the population density, respectively. When the birth and death rates are the same, the population density is at a steady state and is said to be at its carrying capacity. The value at which the two rates are equal will be here referred to as the turnover rate *θ*. The same carrying capacity can be achieved with different birth and death rate functions, and therefore at different corresponding turnover rates (figure 1*a*).

Our approach to study distributions of bacterial filament length places the described density-dependent concepts in the context of a population of cells that can form filamentous individuals. Although prokaryotes had been commonly thought to be in principle immortal [59,60], there is now evidence for programmed cell death and lysis [61], and in addition ageing and senescence [62]. Taking this into account, a multicellular filament is made up of several cells, and a population of filaments can also be considered as a population of cells. In this case, the population size is given by the total number of cells, while the birth and death rates coincide with the birth and death rates of each cell of the filaments. In such a population, one can vary the turnover at which a given fixed carrying capacity is achieved and ask the question if different turnovers correspond to different growth strategies; namely whether during its growth cycle the population is composed mostly of a few long filaments, or of many short filaments. Such a line of questioning is possible because as it will be shown, the rate at which a population reaches its carrying capacity can be different from the rate at which it reaches its stationary filament length distribution. The present work is an attempt to model and experimentally test the dynamics of filamentous bacteria by only considering basic life-history traits of the population. The distribution of filament lengths in the different growth phases can provide insights into the developmental strategies of different filament-forming species, and the population conditions influencing multicellular development.

In the following sections, we first state the model assumptions and we specify the experimental set-up for the culture of five filamentous bacterial species. Since turnover is a key factor in our model, we chose species that also exhibit different turnovers. We hence cultured heterotrophic species with fast generation time and high turnover, and photoautotrophic species characterized by a slower generation time and a low turnover. Heterotrophs differ from photoautotrophs in the way they obtain organic carbon for growth. While the former absorb organic compounds from the environment, the latter autonomously fix carbon using solar light as an energy source. We present the simulation results and test the model through a comparison with our experimental results.

## 2. Methods

### 2.1. Theoretical model

We consider a population of multicellular, undifferentiated, non-branching filamentous bacteria, whose growth is regulated only by the density-dependent birth and death rates. In this context, the total number of cells of the population is used as the population size. The birth and death rates are represented by birth and death probabilities per iteration per-cell. At every iteration, each cell in a filament has a certain probability of dividing into two daughter cells or of dying. Based on these probabilities, the number of cell divisions and cell lyses are calculated in each filament. Accordingly, the filaments are elongated and then broken into smaller pieces. We simulate the change in time of the population until it has stabilized at its carrying capacity. At each iteration, the following steps are performed (see illustration in the electronic supplementary material, §S1).

- Computation of the total number of cells in the population 
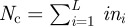
, where *L* is the maximal length of the filaments in the current generation and *n*_*i*_ is the number of filaments of length *i*.
- Computation of the birth and death probabilities per-cell per iteration, corresponding to the birth and death rate functions, respectively *β* = *β*(*N*_c_) and *δ* = *δ* (*N*_c_):2.1
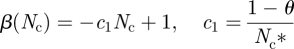
and2.2
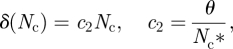
where *N*_c_ is the total number of cells and *N*_c_^*^ the carrying capacity. The latter is defined as the value of the population size, such that *β* (*N*_c_^*^) = *δ*(*N*_c_^*^) = *θ* < 1, where *θ* is the turnover. The birth rate is a decreasing function of *N*_c_. The death rate is an increasing function of *N*_c_.
- For each filament, we compute the number of births *b* and number of deaths *d* occurring in the filament. As *β* and *δ* are the per-cell probabilities in a filament of length *i*, the total number of events *b* or *d* can be viewed as the number of successes in *i* trials, each one with probability of success *β* or *δ*. By picking from a binomial distribution *B*, we determine *b* and *d* for each filament of length *i*:
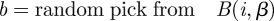
and
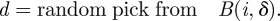
The filament is elongated by *b* cells. Then, a number of *d* cells computed on the basis of the original filament length *i* are randomly selected for lysis in the elongated filament. The filaments resulting from the breakages are stored for the next generation. After completing the scan of all the filaments, the new population is set for the next iteration. The choice of initial conditions and the order at which birth and death are applied to filaments do not significantly affect the results of the simulations. The default initial condition for the simulations is a single cell. Alternative algorithms implementing a reverse or a random order of birth and death steps have been tested and produced similar results (figures not shown).

As stated in point (ii), this model implements linear birth and death rate functions, with fixed carrying capacity. Alternatively, nonlinear functions have been implemented and provided qualitatively comparable results (see the electronic supplementary material, §S2). Considering the difference between those rates, given by *β* − *δ* = −(*c*_1_ + *c*_2_)*N*_c_ + 1, one can observe that the net growth rate (*c*_1_ + *c*_2_) is independent of the turnover *θ*, as *c*_1_ + *c*_2_ = 1/*N*_c_^*^. This implies that regardless of the turnover, the population size is expected to grow with the same curve for any turnover.

### 2.2. Relation between turnover and generation time

Before presenting the results, we provide a formula that helps to understand the connection between the model parameters and the experimental data. In the theoretical model, the turnover is the characterizing property of a strain. However, in natural bacterial populations, turnover is not an easily measurable quantity. For bacteria, doubling time during the exponential growth phase is usually calculated in lieu of generation time. Two populations with the same growth curve also have the same doubling time. Nonetheless, the reality is that the internal dynamics leading to this growth curve can vary. In the same time period, the same concentration of bacteria can be achieved by undertaking many births and deaths, or by conversely having less births and also less deaths. This leads to different turnovers and generation times. Moreover, at carrying capacity, the population density is stable, hence there is no indication of generation time at that stage by measuring doubling time. Here, we calculate the generation time of bacterial populations at carrying capacity in relation to turnover rate.

According to a common approach in ecology to describe the dynamics of an age-structured population [63], the mean age of parents at childbirth in a population with stable age distribution is calculated as:2.3
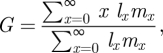
where *x* is the age, *l*_*x*_ is the probability of survival from conception to age *x* and *m*_*x*_ is the mean number of offspring at age *x*. *G* can also be interpreted as a measure of generation time without making any assumption about the growth rate of the population [64]. In our case, age is measured in a number of iterations. We consider bacterial cells as individuals that give birth by cell division and die by a lytic process. Suppose that a cell survives a given iteration with probability 1 − *δ*. Then the probability that a cell is living after *x* iterations if *δ* remains constant is given by *l*_*x*_ = (1 − *δ*)^*x*^. If also the probability of cell division is *β* at every iteration, the average number of offspring at a given age *x* coincides with the probability of birth *β*, whereby *m*_*x*_ = *β*. The generation time of a cell can then be expressed as:2.4
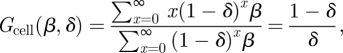
where the last equivalence was obtained by using geometric series. The standard formula in equation (2.3) applies when the growth rate is constant. Given the logistic nature of our model however, the functions *β* and *δ* are actually dependent on the population size *N*_c_. Only when the population approaches the carrying capacity, namely when *N*_c_ → *N*_c_^*^, we have that *β*, *δ* → *θ*, hence the growth rate is constant (equal to 0). We then present a formula for the generation time at the carrying capacity. Substituting *β* = *δ* = *θ* in equation (2.4), the generation time is given by2.5
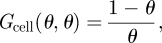
which is a decreasing function of the parameter *θ* (graphical illustrations in the electronic supplementary material, figure S2). Hence, a population with low turnover has a long generation time, whereby cell birth and deaths occur at a low rate. On the other hand, populations with high turnover have a short generation time, whereby births and deaths occur rapidly.

### 2.3. Experimental setting

#### 2.3.1. Bacterial strains

The heterotrophic strains used in the experiments were *Rudanella lutea* DSM 19387^T^ [65] and two new bacteria isolated from a mud sample from tidal flats in Fedderwardersiel, on the North Sea coast of Germany, *Fibrella aestuarina* BUZ 2^T^ [66] and strain *Fibrisoma limi* BUZ 3^T^ [67]. The autotrophic strains were two axenic cyanobacteria, *Nostoc muscorum* SAG 25.82 (identical strains ATCC 27893; PCC 7120) and *Anabaena variabilis* SAG 1403-4b (identical strains CCAP 1403/4B; UTEX 377; ATCC 29211; PCC 6309).

#### 2.3.2. Batch culture experiments for heterotrophs

The growth of the three bacterial species was monitored in batch cultures. Stock bacteria were grown on R2A plates for 3–4 days before starting the experiment. Colonies were homogenized in 0.7 per cent NaCl and finally distributed into 50 ml flasks containing 25 ml of SM (DSMZ 7) or R2A medium (DSMZ 830) for *F. limi*, *R. lutea* and *F. aestuarina*, respectively, reaching a starting optical density (OD) of 0.04. Bacteria were grown on a shaker (120*g*) at 29°C and after different time periods sub-samples were analysed for OD and filament length. For *R. lutea* and *F. aestuarina*, two batch cultures (A and B) were prepared and analysed, whereas for *F. limi* only one batch culture (A) was used for the experiment. Additionally, for each batch, three technical replicates (i.e. a1, a2 and a3) were made. The OD was measured in a spectrophotometer (SpectraMax 384 Plus, Molecular Devices, USA) at 700 and 600 nm. The samples for length measurement were fixed with formaldehyde (final concentration, 2%), 20 µl were distributed on a microscope slide and pictures were taken with a digital camera (Color View, Soft Imaging System, 10X, 20X and 40X objective) connected to a phase-contrast microscope (Olympus BX 51, Germany). For each time point, between 10 and 20 pictures from separate fields were taken and the sizes of the filaments were individually determined by hand using the Soft Imaging software CellF (Olympus, Germany). At least 300 filaments from 10 different pictures were counted for each time point, in order to have a representative distribution of filament lengths.

#### 2.3.3. Batch culture experiments for cyanobacteria

As in the heterotrophic case, the growth of the two cyanobacterial species was monitored in batch cultures. Stock cultures were grown in 20 ml of BG11 medium [68] in 50 ml Erlenmeyers one week prior to the experiment. Two millilitres of grown culture were added to 18 ml of BG11 medium and cultivated on a shaker (150*g*) at room temperature and constant light (670 lux) for three weeks. Every 2 days, 200 µl subsamples were taken, transferred into a microtitre plate and analysed for OD (at 430, 630 and 680 nm; SpectraMax 384 Plus). Filament length was measured by distributing 20 µl samples in a counting chamber Neubauer-improved (Paul Marienfeld GmbH & Co, Germany) and pictures were taken with a digital camera connected to a phase-contrast microscope. Approximately 18 pictures with a 10X magnification objective (or two pictures with a 4X magnification objective) were taken for each time point, and the sizes of the filament were determined using the Soft Imaging software CellF. For each time point, at least 50 filaments were counted in order to have a representative distribution of filament lengths. Three experiment replicates (Erlenmeyer flasks) of each cyanobacteria and four measurement samples of each replicate were analysed for OD. Initial stock cultures were also examined. For filament lengths, four measurement samples were assayed from a single replicate. In figure 3*d*,*e*, we show the results of two experiment replicates (experiments A and B).

To extrapolate the cell number from the filament length, an estimated size of cells forming a filament was measured from several light microscopy pictures. From at least 60 observations, the estimated mean cell lengths are 6.0, 6.7 and 5.9 µm for *R. lutea*, *F. aestuarina* and *F. limi*, respectively. These values are higher than those measured from the inoculum of the same bacteria, indicating that cell size of heterotrophs may change over time. For cyanobacteria, the mean cell length is less variable, and an approximate cell length of 4.0 µm was used for the conversion.

## 3. Results

### 3.1. Simulation results

#### 3.1.1. Turnover and fitness

For the rest of this article, we will refer to the time at which the population reaches its carrying capacity as the first time *t*, such that *N*_c_(*t* + 1)/*N*_c_(*t*) ≤1 + *ε*, where *N*_c_(*t*) is the population size at time *t* and *ε* = 1%. The growth curve of the total number of cells gives information on the fitness of the strains. Figure 1*b* shows that for any turnover, the total number of cells reaches the carrying capacity after less than 20 generations with the same growth curve (average and standard deviation of 1000 runs is shown). In our case, fitness is represented by net growth rate and does not depend on the turnover (§2.1). Hence, populations with different turnovers have the same fitness. On the other hand, when the total number of cells reaches the carrying capacity, the total number of filaments (*N*_f_) differs significantly according to the turnover (figure 1*c*). At this point, *N*_f_ ranges from values close to the carrying capacity, corresponding to almost unicellular filaments (*θ* = 0.9), to values under 20 units (*θ* = 0.001). Figure 1*b*,*c* shows that the steady states of number of cells and number of filaments are not reached at the same time. This is because the stationary filament length distribution is reached much later than the steady state population density (carrying capacity). In the long run, the number of cells per filament reaches a stable equilibrium of about two cells per filament for sufficiently small turnovers, typically less than 0.1 (figure not shown). An analytical support for this result has been obtained by deriving an analogous model in continuous time, as presented in the electronic supplementary material, §S9. For higher turnovers, the effect of discretization leads to a deviation from this equilibrium. Hence, filament length distributions can be affected by turnover rates in temporary phases of the population dynamics, before a stationary distribution is reached. An example is given by the time when the carrying capacity of the population is reached (17 iterations in this case).

#### 3.1.2. Length distribution at the time when carrying capacity is reached

Figure 1*d* shows the box plot of filament lengths when the total number of cells in the population (*N*_c_) has reached the carrying capacity *N*_c_^*^ = 5000 cells. This time point however represents an intermediate stage before a stationary distribution of filament lengths is reached. The corresponding pie-charts indicate the fraction of single cells, double-celled filaments and filaments with more than two cells in the population. When turnover is close to 1 (*θ* = 0.9), the population consists mainly of unicellular and double-celled filaments. Note that *θ* = 0.9 means that at carrying capacity, each cell has a 90 per cent chance of dividing as well as a 90 per cent chance of subsequently dying. When *θ* = 0.1, filaments with one or two cells represent about half of the population. For lower turnovers (*θ* = 0.01, 0.001), the median values of the length are significantly higher. In the latter cases, filaments with more than two cells are more than 90 per cent. Figure 1*d* shows that, as the turnover increases, the fraction of short filaments when the population reaches its carrying capacity also increases. The distribution means at this growth stage were statistically compared by means of a one-way ANOVA and a multiple comparison (see the electronic supplementary material, §S6). The means obtained for *θ* = 0.9 and *θ* = 0.1 were not significantly different. All the other pairwise comparisons were significant.

#### 3.1.3. Length distribution during the transient phase

**Figure 2.**
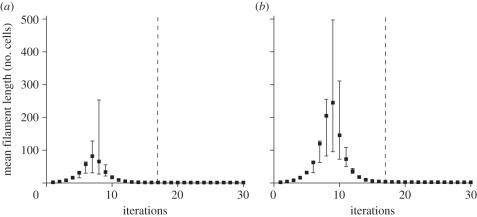
Sample simulations showing the mean filament length along successive iterations for two different turnovers. (*a*) *θ* = 0.9; (*b*) *θ* = 0.1. Squares indicate the mean of the filament lengths obtained through 1000 runs. Edges of the error bars correspond to the 2.5th and 97.5th percentiles of the distribution of the mean filament lengths in the 1000 runs. In both cases, the mean length reaches a peak in the transient phase, and then decreases gradually towards a state comparable to the one of the beginning. Lower turnovers allow for higher transient peaks. The dotted line indicates the iteration at which the carrying capacity is reached.

Figure 2 shows two examples where the mean length of the filaments was calculated every iteration. The plot shows the average of 1000 runs, with error bars indicating the 2.5th and 97.5th percentiles for the mean. The dotted line indicates the iteration at which the total number of cells reaches the carrying capacity. An increase to a peak of the mean length followed by a decay towards a steady state is observed in all cases. At the end of the decreasing phase, the mean length is comparable to that in the beginning. The same pattern was also observed for *θ* = 0.01, 0.001 (see the electronic supplementary material, §S3). An analytical characterization of the filament length dynamics has been carried out in the electronic supplementary material (§S8), where an approximation for the iteration of first filament breakage has been derived. In order to statistically analyse the average length trend, we considered three reference time points and we compared the distribution of the means at those points using a non-parametric hypothesis test. As reference points we chose the starting point, the peak and the carrying capacity. The outcome of the tests provides statistical evidence of a significant increase and subsequent decrease of the average length. Details and results are given in the electronic supplementary material, §S6.

Significant differences are found in the maximum average length achieved. Long filaments, with a mean length up to 250 cells, are observed at a turnover of *θ* = 0.1. Longer filaments can be achieved with lower turnovers (electronic supplementary material, figure S4). For a higher turnover (*θ* = 0.9), the average length of filaments at its peak does not exceed 100 cells. The observed trend indicates the prevalence of a cycle in the mean length induced by the birth and death rates, whereby filaments are short in the beginning, longer in the transient phase and again short at the carrying capacity. Moreover, in the transient phase, filaments with low turnover can elongate more than filaments with high turnover.

### 3.2. Experimental results and model validation

In the theoretical model, the population stays at the carrying capacity once this has been reached. However, as bacterial strains were not cultured in a chemostat, carrying capacities could not be maintained indefinitely. In order to assure that the experimental dataset and the simulation data are comparable, we indicate with a dotted line the time point where the bacterial populations reach their carrying capacity on each plot of the experimental results. This point was determined from the OD curves (not shown). Subsequent to reaching the carrying capacity, the process of filament breakage is eventually accelerated in the empirical case owing to a progressive decrease in the available nutrients.

In order to prove the significant increase and decrease of bacterial filament length, we performed an ANOVA test associated with a multiple comparison on the data based on Tukey's HSD test [69]. Details of the tests for each species and within species are provided in the electronic supplementary material, §S6.

In the following sections, the time point 0 is represented by the inoculum. The starting culture of heterotrophs was taken from plate colonies and vortexed. Because of the nutrient depletion in plate colonies and mechanical breakage through vortexing, the heterotrophic inoculum is composed mainly of single cells. The starting cultures of cyanobacteria were taken from cultures grown for more than one week, gently pipetted and diluted. At inoculum, cyanobacteria filaments had an average filament length of about 45 cells (*N. muscorum*) and about 75 cells (*A. variabilis*).

#### 3.2.1. Mean length trend

**Figure 3.**
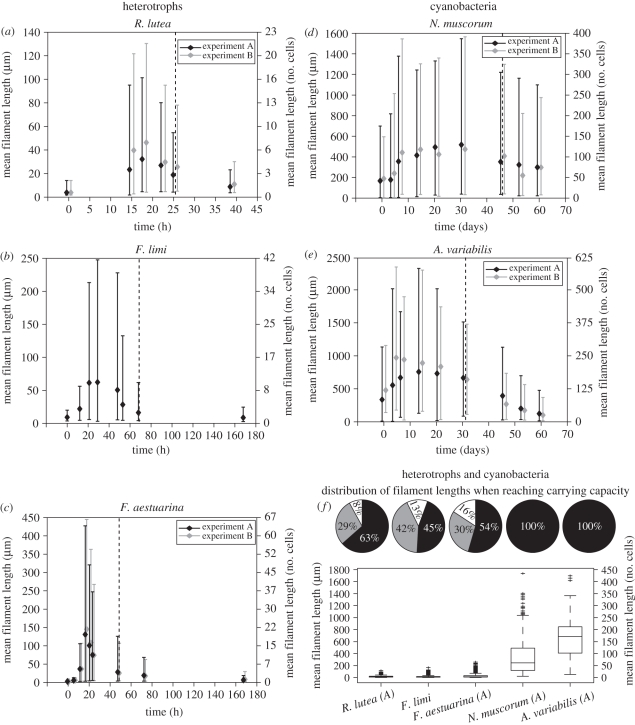
(*a*)–(*e*) Empirical measurements showing mean length of bacteria against time. Edges of the error bars correspond to the 2.5th and 97.5th percentiles of the measured lengths. Diamonds indicate the mean of measured filament length at each time point. Data from both heterotrophic (*a*–*c*) and photoautotrophic (*d*–*e*) species are shown. The zero time point corresponds to the inoculum. The dotted line indicates the time at which the carrying capacity was reached. (*f*) Box plots of filament lengths when the bacterial populations reach their carrying capacity. The capital letter next to the species name indicates the experiment used in the plot. The corresponding pie charts on the top indicate the proportion of single-celled (white), two-celled (grey) and longer (black) filaments in the population.

**Figure 4.**
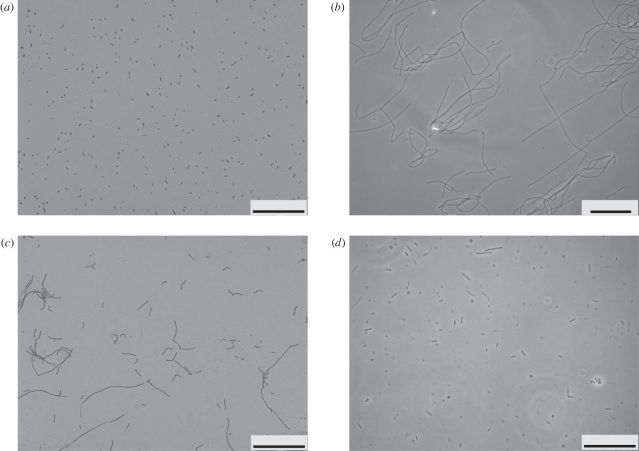
Bright field micrographs of *F. aestuarina* bacteria at different stages of their growth. (*a*) The initial population (inoculum) is composed of many small filaments. (*b*) After 17 h, the filaments reach their maximal length. (*c*) After this peak, the bacteria progressively break towards a mixed population of short and medium filaments as they reach their carrying capacity (48 h). (*d*) Eventually, the filament lengths approach the mean of the inoculum (168 h). Scale bars: (*a*,*d*) 50 µm; (*b*,*c*) 100 µm.

Figure 3*a*–*c* show the change in time of the mean length of heterotrophic bacteria *R. lutea*, *F. limi* and *F. aestuarina*. The pattern is similar in all three cases: the mean length increases until a peak, after which it decreases and reaches a value approaching that of the inoculum (see the electronic supplementary material, table S1). This trend is recognizable in figure 4, showing micrographs of *F. aestuarina* taken at different time points. Remarkably, experiments A and B on *F. aestuarina* give very similar results, showing a high repeatability of the observed pattern (figure 3*c*). A comparison of the theoretical and *F. aestuarina* length distributions against time is shown in the electronic supplementary material (§S4 and figure S6). The mean length of cyanobacteria, tracked for two months, shows a qualitative trend similar to that of heterotrophs, albeit much slower. Both the *A. variabilis* and *N. muscorum* species have a mean-length peak in the transient phase. However, because of the low turnover, they need more time than heterotrophs to reach the stationary distribution, as predicted by the model. When they reach the carrying capacity, they are still long.

The pattern observed in heterotrophs is comparable with the simulation results shown in figure 2. The trend of the cyanobacteria, although not so markedly conclusive, gives an indication that the model predictions also hold in autotrophs. An additional illustration of the mean length trend of the cultured species is provided in the electronic supplementary material, figure S5.

#### 3.2.2. Length in the transient phase

Generation times in heterotrophs and cyanobacteria are very different. While the former are observed on the scale of hours, the latter grow on a scale of days/weeks. The difference could be, in principle, owing to the different trophic state of the bacteria. This hypothesis could be tested by means of a comparison between different generation times intraspecifically in the autotrophs or in the heterotrophs. However, this goes beyond the scope of the present work and we proceed relying on the mathematical derivation of §2.2. According to the relationship between generation time and turnover established in §2.2, we assume that the heterotrophs and cyanobacteria have high and low turnovers, respectively. The simulation results presented in §3.1.3 show that the peak value of the mean length in the transient phase increases with decreasing turnover. Figure 3 shows that heterotrophic filaments will rarely be longer than 60 cells. The length of cyanobacteria can be significantly higher than that of heterotrophs. *Nostoc muscorum* and *A. variabilis* filaments have up to around 350 and 600 cells, respectively. Hence, cultured bacterial populations support the model predictions, indicating that bacteria with a putatively higher turnover (in this case heterotrophic species) are able to elongate less than those with lower turnover (cyanobacteria).

#### 3.2.3. Distribution of lengths at the time when carrying capacity is reached

In figure 3*f*, the box plots and the pie charts show the proportion of short and long filaments in the cultured bacterial populations when they reach their carrying capacity. As for the simulation results, the pie charts illustrate three length classes, namely single cells, double-celled filaments and filaments with more than two cells. The heterotrophs show a considerable amount of short filaments, with a proportion of single cells and double-celled filaments varying across species in the range 8–16% and 29–42%, respectively. In cyanobacteria, filaments with one or two cells are absent. Figure 3*f* can be compared with figure 1*d*. Again, the experimental results confirm the theoretical predictions, according to which short filaments at the carrying capacity are more abundant in populations with high turnover.

#### 3.2.4. Serial transfer experiment and simulations

**Figure 5.**
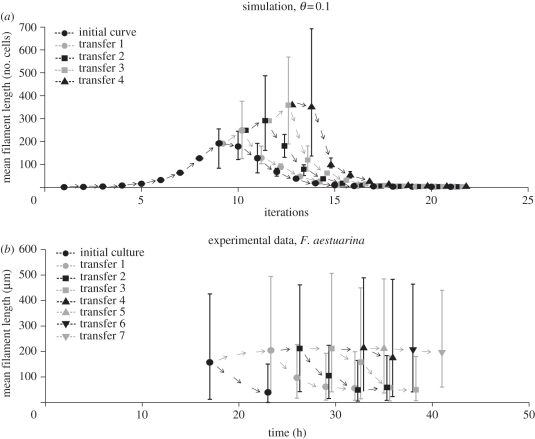
Simulations and experiment of serial transfers. Symbol shapes and shading indicate different transfers. Arrows indicate the trend of each culture. (*a*) Computer simulations of successive transfers. When the average filament length is at its peak, the population density is below the carrying capacity. Whenever a culture reaches this peak, the corresponding average length is used as a starting condition of a new simulation at lower density (transfers 1–4). This procedure simulates the transfer of an aliquot of a higher density population to a fresh medium. Edges of the error bars correspond to the 2.5th and 97.5th percentiles of the distribution of the mean filament lengths in the 1000 runs (black circles, initial curve; grey circles, transfer 1; black squares, transfer 2; grey squares, transfer 3; black triangles, transfer 4). (*b*) Average length of *F. aestuarina* during serial transfers to fresh medium. The first transfer was done after 17 h. For the first five transferred cultures, the mean length was measured and plotted at several successive time points. For subsequent cultures, only one measurement was taken. Edges of the error bars correspond to the 2.5th and 97.5th percentiles of the measured lengths.

If filament elongation is governed by density-dependent processes as hypothesized in the model in §2.1, then interventions on such underlying processes should produce testable hypotheses. Experimental manipulation of cell densities is one simple possibility. If the population drops significantly below its carrying capacity, the filaments that survive in this new condition should be able to elongate or at least maintain their original length until the carrying capacity is reached again. We simulated and experimentally carried out a series of successive transfers of bacterial populations, whereby a fraction of the population was transferred to a fresh medium whenever the average length of the original population was close to its peak. Filaments developing in the fresh medium were expected to keep or increase the length reached at the previous stage. Details of the computational and experimental settings are provided in the electronic supplementary material, §S7. Figure 5*a* shows the average length of filaments obtained by successive transfer simulations. Figure 5*b* shows the average length of *F. aestuarina* tracked along successive transfers. In this case, filament size slightly increases or remains almost constant in most of the cases. The fact that filaments do not elongate so strongly as in the simulations could be owing to physical handling (breakage of long filaments while pipetting) or to other culture conditions influencing the elongation that were not considered in the model. The model results indicate that the filaments transferred to the fresh medium maintain or increase their length, and eventually, start shortening when they reach their carrying capacity again. The experimental results, although not as strong as the simulations, show that the transferred bacteria are generally able to maintain their length.

## 4. Discussion

Filamentous bacteria can be viewed as a simple example of multicellular individuals. Some of them, such as filamentous cyanobacteria, have been present in the environment since the early evolution of life, as indicated by fossil records and phylogenetic analyses [70–73]. The theoretical model shows that at various life stages, two populations with the same fitness and carrying capacity can differ in the composition of filament length classes according to the life-history traits regulating their growth. Although the long-term stationary distribution of filament length is the same for any sufficiently small turnover rate, the differences owing to turnover observed at other growth stages (e.g. transient phase, when reaching carrying capacity) are quantitatively significant and biologically relevant. Filaments belonging to a population with high turnover (and hence short generation time) have moderate lengths at their maximum growth phase. Furthermore, when the population reaches carrying capacity, they are chiefly unicellular. Individuals of a population with low turnover (and hence long generation time) can instead become very long in the transient phase, and still be multicellular when reaching the carrying capacity. The species that we cultured show evidence that the predictions of the model hold. The relationship between bacterial generation time and turnover derived in §2.2 helps us to understand the relationship between simulations and experiments. The heterotrophs have a faster generation time than the cyanobacteria (and hence a higher turnover). As a consequence, while the heterotrophic filaments are rarely longer than 60 cells, the cyanobacteria can reach lengths of up to 600 cells. Both theory and experiments show that according to different life-history traits, bacteria can cover a wide spectrum of lengths, indicating that multicellularity can be achieved to different degrees. Moreover, a key feature of this model is that the predominance of multicellularity during the life cycle is not necessarily determined by morphology-dependent differences in fitness. Here, the factors affecting multicellularity are birth and death rates, and hence turnover rate and generation time.

The model indicates a common temporal behaviour for filament length distributions. The mean length increases and peaks during the transient growth phase, and then decreases until it reaches a value approaching the initial conditions. The results from cultured bacterial populations of heterotrophic and photoautotrophic species show that the predicted trend in filament length distribution is robust across species, and is representative of what occurs in nature. The fact that individuals eventually become shorter (and of average length 2 at the stationary distribution) regardless of the turnover is because of the effect of cell lysis on a filament. Consider a filament of length *L*. A cell division (birth) increases its length by one unit, namely the filament length becomes *L* + 1. By contrast, after a cell lysis, the expected filament length will be on average *L*/2. The average effect of cell death on length is hence much larger than that of cell birth. This is consistent with the expectation that the average filament length in a population can keep increasing if the death rate remains sufficiently low, as shown in the estimation of the time of first filament split (electronic supplementary material, §8). When the average filament length in the population is at its stationary distribution, cell birth and death have the same effect. This is achievable only if the filaments are of average length 2. This argument is supported by the analysis of the analogous continuous time model derived in the electronic supplementary material. In this context, a way to avoid the breakage into small filaments is to decrease the effect of cell lysis on organism size. A solution in this direction is the evolution of a second or third dimension, as in spherical multicellular bacteria [74] or microbial biofilms. In this case, if we think of a square or a ball of *N* cells, a cell division will still lead to an individual of *N* + 1 cells, but cell death will also only decrease size by one unit, resulting in a size of *N* − 1 (as opposed to *N*/2).

There are, to our knowledge, no previous theoretical and experimental results that are directly comparable to the results presented here. However, the extensive work of Hahn & Hoefle [27] and Hahn *et al*. [45] set important precedents and reference points. These works focus on the effects of predators on selection for longer filament lengths. Their results are not directly comparable to those presented here because the death rates owing to predators preferentially afflict smaller filaments and single cells. However, they also provide revealing information on the dynamics of filament formation in the absence of predators. Hahn *et al*. [45] show that increasing the nutrient flow and the dilution rate of a chemostat in the absence of predators leads to longer filament lengths. This corresponds to our prediction that in transient growth conditions with lower population densities, filament lengths would get longer. Furthermore, although they do not present extensive data on this, [45] they mention that in batch culture, the percentage of multicellular filaments is higher in the exponential phase in comparison with the stationary phase. This situation is again consistent with the predictions presented here.

The model studied here is a general model of filamentous growth, and is not restricted to prokaryotes. Many simple forms of eukaryotic algae grow in a multicellular filamentous form, and the theoretical results presented here are in principle also valid for such eukaryotes. However, it is clear that eukaryotic algae have surpassed their prokaryotic counterparts in their morphological diversity. There are at least six major algal lineages in which filamentous multicellularity has evolved: the Phaeophyta, Rhodophyta, Chrysophyta, Chlorophyta, Charophyta and Embryophyta [75]. However, in each of the latter cases, higher dimensional variants have also evolved, whereupon cell division can occur in more than one planar dimension. This diversity can for example range from the simple globular multicellularity of some of the Volvocales (within the Cholorphyta), to the highly differentiated and developmentally complex vascular land plants (within the Embryophyta). Furthermore, plants can differentiate into diverse developmental modules such as branching nodes, leaves, inflorescences and root structures. By combining such modules in different combinations, they can access a wider variety of morphologies during their growth process [76,77]. This flexibility in morphology can translate into both phenotypic plasticity and evolvability.

Developmental life cycles are not unique to multicellular eukaryotes, and an understanding of how they evolved is lacking. Theory and experiments in this article indicate that the size of the individual, in terms of number of cells, can follow a cycle: from unicellular (or short length) to multicellular and back to unicellular (or short length) again. Each time, the initiation of the cycle can be hypothetically achieved by moving cell density below the carrying capacity (e.g. increases in fluid or nutrient availability). The transfer experiment and simulations do not contradict this hypothesis.

In our model, only the birth and death rates play a role in the change in size during the bacterial life cycle. These life-history traits are intrinsic properties of every living organism. This pattern, which automatically arises from the interplay between ecology and the filamentous nature of the bacteria, is an emergent property. As such, it can serve as the basis for a primitive life cycle, upon which a more complex developmental programme can be subsequently built. As a case in point, some species of *Nostoc* can differentiate into hormogonia during low growth phases characterized by high stress or scarce resources. These hormogonia are short filaments specialized for survival in harsh conditions, which subsequently migrate to a new area before growing into full-grown filaments again [78]. Our results suggest that the cycling of filament lengths between growth and static phases of the population is a pre-existing context within which multicellular developmental cycles and differentiation can evolve.

## References

[1] Buss L. W. 1987 The evolution of individuality. Princeton, NJ: Princeton University Press

[2] Bonner J. T. 1998 The origins of multicellularity. Integr. Biol. 1, 27–36 10.1002/(SICI)1520-6602(1998)1:1%3C27::AID-INBI4%3E3.0.CO;2-6 (doi:10.1002/(SICI)1520-6602(1998)1:1<27::AID-INBI4>3.0.CO;2-6)

[3] Carroll S. B. 2001 Chance and necessity: the evolution of morphological complexity and diversity. Nature 409, 1102–1109 10.1038/35059227 (doi:10.1038/35059227) 11234024

[4] King N. 2004 The unicellular ancestry of animal development. Dev. Cell 7, 313–325 10.1016/j.devcel.2004.08.010 (doi:10.1016/j.devcel.2004.08.010) 15363407

[5] Grosberg R. K. Strathmann R. R. 2007 The evolution of multicellularity: a minor major transition? Ann. Rev. Ecol. Evol. Syst. 38, 621–654 10.1146/annurev.ecolsys.36.102403.114735 (doi:10.1146/annurev.ecolsys.36.102403.114735)

[6] Rokas A. 2008 The origins of multicellularity and the early history of the genetic toolkit for animal development. Ann. Rev. Genet. 42, 235–251 10.1146/annurev.genet.42.110807.091513 (doi:10.1146/annurev.genet.42.110807.091513) 18983257

[7] Smith J. M. Szathmáry E. 1995 The major transitions in evolution. Oxford, UK: Freeman

[8] Michod R. E. 2000 Darwinian dynamics: evolutionary transitions in fitness and individuality. Princeton, NJ: Princeton University Press

[9] Buss L. W. 1982 Somatic-cell parasitism and the evolution of somatic tissue compatibility. Proc. Natl Acad. Sci. USA 79, 5337–5341 10.1073/pnas.79.17.5337 (doi:10.1073/pnas.79.17.5337) 6957867 PMC346891

[10] Strassmann J. E. Zhu Y. Queller D. C. 2000 Altruism and social cheating in the social amoeba *Dictyostelium discoideum*. Nature 408, 965–967 10.1038/35050087 (doi:10.1038/35050087) 11140681

[11] Velicer G. J. Kroos L. Lenski R. E. 2000 Developmental cheating in the social bacterium *Myxococcus xanthus*. Nature 404, 598–601 10.1038/35007066 (doi:10.1038/35007066) 10766241

[12] Rainey P. B. Rainey K. 2003 Evolution of cooperation and conflict in experimental bacterial populations. Nature 425, 72–74 10.1038/nature01906 (doi:10.1038/nature01906) 12955142

[13] Griffin A. S. West S. A. Buckling A. 2004 Cooperation and competition in pathogenic bacteria. Nature 430, 1024–1027 10.1038/nature02744 (doi:10.1038/nature02744) 15329720

[14] Ackermann M. Stecher B. Freed N. E. Songhet P. Hardt W. D. Doebeli M. 2008 Self-destructive cooperation mediated by phenotypic noise. Nature 454, 987–990 10.1038/nature07067 (doi:10.1038/nature07067) 18719588

[15] Dworkin M. 1972 The myxobacteria: new directions in studies of procaryotic development. Crit. Rev. Microbiol. 1, 435–452 10.3109/10408417209103873 (doi:10.3109/10408417209103873)

[16] Bonner J. T. 1974 On development: the biology of form. Cambridge, MA: Harvard University Press

[17] Rosenberg E. Keller K. H. Dworkin M. 1977 Cell density-dependent growth of *Myxococcus xanthus* on casein. J. Bacteriol. 129, 770–777 402357 10.1128/jb.129.2.770-777.1977 PMC235010

[18] Pfeiffer T. Bonhoeffer S. 2003 An evolutionary scenario for the transition to undifferentiated multicellularity. Proc. Natl Acad. Sci. USA 100, 1095–1098 10.1073/pnas.0335420100 (doi:10.1073/pnas.0335420100) 12547910 PMC298732

[19] Kreft J. U. 2004 Biofilms promote altruism. Microbiology 150, 2751–2760 10.1099/mic.0.26829-0 (doi:10.1099/mic.0.26829-0) 15289571

[20] Solari C. A. Ganguly S. Kessler J. O. Michod R. E. Goldstein R. E. 2006 Multicellularity and the functional interdependence of motility and molecular transport. Proc. Natl Acad. Sci. USA 103, 1353–1358 10.1073/pnas.0503810103 (doi:10.1073/pnas.0503810103) 16421211 PMC1360517

[21] Berleman J. E. Kirby J. R. 2009 Deciphering the hunting strategy of a bacterial wolfpack. FEMS Microbiol. Rev. 33, 942–957 10.1111/j.1574-6976.2009.00185 (doi:10.1111/j.1574-6976.2009.00185) 19519767 PMC2774760

[22] Solari C. A. Kessler J. O. Michod R. E. 2006 A hydrodynamics approach to the evolution of multicellularity: flagellar motility and germ-soma differentiation in volvocalean green algae. Am. Nat. 167, 537–554 10.1086/501031 (doi:10.1086/501031) 16670996

[23] Willensdorfer M. 2009 On the evolution of differentiated multicellularity. Evolution 63, 306–323 10.1111/j.1558-5646.2008.00541.x (doi:10.1111/j.1558-5646.2008.00541.x) 19154376

[24] Stanley S. M. 1973 An ecological theory for the sudden origin of multicellular life in the late precambrian. Proc. Natl Acad. Sci. USA 70, 1486–1489 10.1073/pnas.70.5.1486 (doi:10.1073/pnas.70.5.1486) 16592084 PMC433525

[25] Guede H. 1979 Grazing by protozoa as selection factor for activated sludge bacteria. Microb. Ecol. 5, 225–237 10.1007/BF02013529 (doi:10.1007/BF02013529) 24232496

[26] Shikano S. Luckinbill L. S. Kurihara Y. 1990 Changes of traits in a bacterial population associated with protozoal predation. Microb. Ecol. 20, 75 10.1007/BF02543868 (doi:10.1007/BF02543868) 24193965

[27] Hahn M. W. Hoefle M. G. 1998 Grazing pressure by a bacterivorous flagellate reverses the relative abundance of *Comamonas acidovorans* PX54 and *vibrio* strain CB5 in chemostat cocultures. Appl. Environ. Microbiol. 64, 1910–1918 9572971 10.1128/aem.64.5.1910-1918.1998 PMC106250

[28] Pernthaler J. Zollner E. Warnecke F. Jurgens K. 2004 Bloom of filamentous bacteria in a mesotrophic lake: identity and potential controlling mechanism. Appl. Environ. Microbiol. 70, 6272–6281 10.1128/AEM.70.10.6272-6281.2004 (doi:10.1128/AEM.70.10.6272-6281.2004) 15466575 PMC522086

[29] Shapiro J. A. 1998 Thinking about bacterial populations as multicellular organisms. Ann. Rev. Microbiol. 52, 81–104 10.1146/annurev.micro.52.1.81 (doi:10.1146/annurev.micro.52.1.81) 9891794

[30] Whitton B. A. Editors M. P. 2000 The ecology of cyanobacteria: their diversity in time and space. Berlin, Germany: Springer

[31] Paerl H. W. Pinckney J. L. Steppe T. F. 2000 Cyanobacterial-bacterial mat consortia: examining the functional unit of microbial survival and growth in extreme environments. Environ. Microbiol. 2, 11–26 10.1046/j.1462-2920.2000.00071.x (doi:10.1046/j.1462-2920.2000.00071.x) 11243256

[32] Tannock G. W. 1999 Analysis of the intestinal microflora: a renaissance. *Antonie van Leeuwenhoek*. Int. J. Gen. Mol. Microbiol. 76, 265–278 10.1023/A:1002038308506 (doi:10.1023/A:1002038308506) 10532383

[33] Rosen D. A. Hooton T. M. Stamm W. E. Humphrey P. A. Hultgren S. J. 2007 Detection of intracellular bacterial communities in human urinary tract infection. PLoS Med. 4, 1949–1958 10.1371/journal.pmed.0040329 (doi:10.1371/journal.pmed.0040329) PMC2140087 18092884

[34] Kahru M. Horstmann U. Rud O. 1994 Satellite detection of increased cyanobacteria blooms in the Baltic Sea: natural fluctuation or ecosystem change. AMBIO 23, 469–472

[35] Sellner K. G. 1997 Physiology, ecology, and toxic properties of marine cyanobacteria blooms. Limnol. Oceanogr. 42, 1089–1104 10.4319/lo.1997.42.5_part_2.1089 (doi:10.4319/lo.1997.42.5_part_2.1089)

[36] Ramothokang T. R. Drysdale G. D. Bux F. 2003 Isolation and cultivation of filamentous bacteria implicated in activated sludge bulking. Water SA 29, 405–410

[37] Nielsen P. H. Kragelund C. Seviour R. J. Nielsen J. L. 2009 Identity and ecophysiology of filamentous bacteria in activated sludge. FEMS Microbiol. Rev. 33, 969–998 10.1111/j.1574-6976.2009.00186.x (doi:10.1111/j.1574-6976.2009.00186.x) 19622067

[38] Stanier R. Y. Cohenbazire G. 1977 Phototropic prokaryotes: cyanobacteria. Ann. Rev. Microbiol. 31, 225–274 10.1146/annurev.mi.31.100177.001301 (doi:10.1146/annurev.mi.31.100177.001301) 410354

[39] Wolk C. P. 1996 Heterocyst formation. Annu. Rev. Genet. 30, 59–78 10.1146/annurev.genet.30.1.59 (doi:10.1146/annurev.genet.30.1.59) 8982449

[40] Rossetti V. Schirrmeister B. E. Bernasconi M. V. Bagheri H. C. 2010 The evolutionary path to terminal differentiation and division of labor in cyanobacteria. J. Theoret. Biol. 262, 23–34 10.1016/j.jtbi.2009.09.009 (doi:10.1016/j.jtbi.2009.09.009) 19761779

[41] Jurgens K. Arndt H. Rothhaupt K. O. 1994 Zooplankton-mediated changes of bacterial community structure. Microb. Ecol. 27, 27–42 10.1007/BF00170112 (doi:10.1007/BF00170112) 24190166

[42] Pernthaler J. Posch T. Simek K. Vrba J. Amann R. Psenner R. 1997 Contrasting bacterial strategies to coexist with a flagellate predator in an experimental microbial assemblage. Appl. Environ. Microbiol. 63, 596–601 16535516 10.1128/aem.63.2.596-601.1997 PMC1389522

[43] Pernthaler J. 2005 Predation on prokaryotes in the water column and its ecological implications. Nat. Rev. Microbiol. 3, 537–546 10.1038/nrmicro1180 (doi:10.1038/nrmicro1180) 15953930

[44] Wu Q. L. Boenigk J. Hahn M. W. 2004 Successful predation of filamentous bacteria by a nanoflagellate challenges current models of flagellate bacterivory. Appl. Environ. Microbiol. 70, 332–339 10.1128/AEM.70.1.332-339.2004 (doi:10.1128/AEM.70.1.332-339.2004) 14711660 PMC321292

[45] Hahn M. W. Moore E. R. Hoefle M. G. 1999 Bacterial filament formation, a defense mechanism against flagellate grazing, is growth rate controlled in bacteria of different phyla. Appl. Environ. Microbiol. 65, 25–35 9872755 10.1128/aem.65.1.25-35.1999 PMC90978

[46] Kamp A. Roy H. Schulz-Vogt H. N. 2008 Video supported analysis of Beggiatoa filament growth, breakage, and movement. Microb. Ecol. 56, 484–491 10.1007/s00248-008-9367-x (doi:10.1007/s00248-008-9367-x) 18335158 PMC2755761

[47] Wu H. Gao K. Villafane V. E. Watanabe T. Helbling E. W. 2005 Effects of solar UV radiation on morphology and photosynthesis of filamentous cyanobacterium *Arthrospira platensis*. Appl. Environ. Microbiol. 71, 5004–5013 10.1128/AEM.71.9.5004-5013.2005 (doi:10.1128/AEM.71.9.5004-5013.2005) 16151080 PMC1214621

[48] Kruskopf M. 2006 Growth and filament length of the bloom forming *Oscillatoria simplicissima* (oscillatoriales, cyanophyta) in varying N and P concentrations. Hydrobiologia 556, 357–362 10.1007/s10750-005-1061-0 (doi:10.1007/s10750-005-1061-0)

[49] Lehtimaki J. Moisander P. Sivonen K. Kononen K. 1997 Growth, nitrogen fixation, and nodularin production by two Baltic Sea cyanobacteria. Appl. Environ. Microbiol. 63, 1647–1656 16535588 10.1128/aem.63.5.1647-1656.1997 PMC1389143

[50] Nobel W. T. D. Matthijs H. C. P. Elert E. V. Mur L. R. 1998 Comparison of the light-limited growth of the nitrogen-fixing cyanobacteria *Anabaena* and *Aphanizomenon*. New Phytol. 138, 579–587 10.1046/j.1469-8137.1998.00155.x (doi:10.1046/j.1469-8137.1998.00155.x)

[51] Sigee D. C. Glenn R. Andrews M. J. Bellinger E. G. Butler R. D. Epton H. A. S. Hendry R. D. 1999 Biological control of cyanobacteria: principles and possibilities. Hydrobiologia 395, 161–172 (Conference on Hydrobiologia, Leicester, England, March 1996.) 10.1023/A:1017097502124 (doi:10.1023/A:1017097502124)

[52] Van Hannen E. J. Zwart G. Van Agterveld M. P. Gons H. J. Ebert J. Laanbroek H. J. 1999 Changes in bacterial and eukaryotic community structure after mass lysis of filamentous cyanobacteria associated with viruses. Appl. Environ. Microbiol. 65, 795–801 9925618 10.1128/aem.65.2.795-801.1999 PMC91097

[53] Weinbauer M. G. 2004 Ecology of prokaryotic viruses. FEMS Microbiol. Rev. 28, 127–181 10.1016/j.femsre.2003.08.001 (doi:10.1016/j.femsre.2003.08.001) 15109783

[54] Daft M. J. Stewart W. D. P. 1973 Light and electron-microscope observations on algal lysis by bacterium CP-1. New Phytol. 72, 799–808 10.1111/j.1469-8137.1973.tb02055.x (doi:10.1111/j.1469-8137.1973.tb02055.x)

[55] Lewis K. 2000 Programmed death in bacteria. Microbiol. Mol. Biol. Rev. 64, 503–514 10.1128/MMBR.64.3.503-514.2000 (doi:10.1128/MMBR.64.3.503-514.2000) 10974124 PMC99002

[56] Ning S. B. Guo H. L. Wang L. Song Y. C. 2002 Salt stress induces programmed cell death in prokaryotic organism *Anabaena*. J. Appl. Microbiol. 93, 15–28 10.1046/j.1365-2672.2002.01651.x (doi:10.1046/j.1365-2672.2002.01651.x) 12067370

[57] Berman-Frank I. Bidle K. D. Haramaty L. Falkowski P. G. 2004 The demise of the marine cyanobacterium, *Trichodesmium* spp., via an autocatalyzed cell death pathway. Limnol. Oceanogr. 49, 997–1005 10.4319/lo.2004.49.4.0997 (doi:10.4319/lo.2004.49.4.0997)

[58] Adamec F. Kaftan D. Nedbal L. 2005 Stress-induced filament fragmentation of *Calothrix elenkinii* (cyanobacteria) is facilitated by death of high-fluorescence cells. J. Phycol. 41, 835–839 10.1111/j.1529-8817.2005.00104.x (doi:10.1111/j.1529-8817.2005.00104.x)

[59] Williams G. C. 1957 Pleiotropy, natural-selection, and the evolution of senescence. Evolution 11, 398–411 10.2307/2406060 (doi:10.2307/2406060)

[60] Rose M. R. 1991 Evolutionary biology of aging. Oxford, UK: Oxford University Press

[61] Rice K. C. Bayles K. W. 2008 Molecular control of bacterial death and lysis. Microbiol. Mol. Biol. Rev. 72, 85–109 10.1128/MMBR.00030-07 (doi:10.1128/MMBR.00030-07) 18322035 PMC2268280

[62] Ackermann M. Stearns S. C. Jenal U. 2003 Senescence in a bacterium with asymmetric division. Science 300, 1920 10.1126/science.1083532 (doi:10.1126/science.1083532) 12817142

[63] Leslie P. H. 1945 On the use of matrices in certain population mathematics. Biometrika 33, 183–212 10.1093/biomet/33.3.183 (doi:10.1093/biomet/33.3.183) 21006835

[64] Charlesworth B. 1994 Evolution in age-structured populations, 2nd edn. Cambridge, UK: Cambridge University Press 10.1017/CBO9780511525711 (doi:10.1017/CBO9780511525711)

[65] Weon H. Y. Noh H. J. Son J. A. Jang H. B. Kim B. Y. Kwon S. W. Stackebrandt E. 2008 *Rudanella lutea* gen. nov., sp. nov., isolated from an air sample in Korea. Int. J. Syst. Evol. Microbiol. 58, 474–478 10.1099/ijs.0.65358-0 (doi:10.1099/ijs.0.65358-0) 18218952

[66] Filippini M. Svercel M. Laczko E. Kaech A. Ziegler U. Bagheri H. C. 2011 *Fibrella aestuarina* gen. nov., sp. nov., a filamentous bacterium of the family cytophagaceae isolated from North Sea tidal flats and emended description of the genus *Rudanella* Weon *et al.*, 2008. Int. J. Syst. Evol. Microbiol. 61, 184–189 10.1099/ijs.0.020503-0 (doi:10.1099/ijs.0.020503-0) 20190021

[67] Filippini M. Kaech A. Ziegler U. Bagheri H. C. In press *Fibrisoma limi* gen. nov., sp. nov., a filamentous bacterium isolated from tidal flats. Int. J. Syst. Evol. Microbiol. (doi:10.1099/ijs.0.025395-0) 10.1099/ijs.0.025395-0 20601484

[68] Rippka R. DeReuelles J. Waterbury J. B. Herdman M. Stanier R. Y. 1979 Generic assignments, strain histories and properties of pure cultures of cyanobacteria. J. Gen. Microbiol. 111, 1–61

[69] Tukey J. W. 1949 Comparing individual means in the analysis of variance. Biometrics 5, 99–114 10.2307/3001913 (doi:10.2307/3001913) 18151955

[70] Hofmann H. J. 1976 Precambrian Microflora, Belcher Islands, Canada: significance and systematics. J. Paleontol. 50, 1040–1073

[71] Amard B. Bertrand-Sarfati J. 1997 Microfossils in 2000 Ma old cherty stromatolites of the Franceville Group, Gabon. Precambr. Res. 81, 197–221 10.1016/S0301-9268(96)00035-6 (doi:10.1016/S0301-9268(96)00035-6)

[72] Westall F. de Ronde C. E. J. Southam G. Grassineau N. Colas M. Cockell C. S. Lammer H. 2006 Implications of a 3.472-3.333 Gyr-old subaerial microbial mat from the Barberton greenstone belt, South Africa for the UV environmental conditions on the early Earth. Phil. Trans. R. Soc. B 361, 1857–1875 17008224 10.1098/rstb.2006.1896 PMC1664690

[73] Schirrmeister B. E. Antonelli A. Bagheri H. C. 2011 The origin of multicellularity in cyanobacteria. BMC Evol. Biol. 11, 45 10.1186/1471-2148-11-45 (doi:10.1186/1471-2148-11-45) 21320320 PMC3271361

[74] Keim C. N. Martins J. L. Abreu F. Rosado A. S. de Barros H. L. Borojevic R. Lins U. Farina M. 2004 Multicellular life cycle of magnetotactic prokaryotes. FEMS Microbiol. Lett. 240, 203–208 10.1016/j.femsle.2004.09.035 (doi:10.1016/j.femsle.2004.09.035) 15522508

[75] Niklas K. 2000 The evolution of plant body plans: a biomechanical perspective. Ann. Bot. 85, 411–438 10.1006/anbo.1999.1100 (doi:10.1006/anbo.1999.1100)

[76] Prusinkiewicz P. Lindenmayer A. 1990 The algorithmic beauty of plants. New York, NY: Springer

[77] Sultan S. 2000 Phenotypic plasticity for plant development, function and life history. Trends Plant Sci. 5, 537–542 10.1016/S1360-1385(00)01797-0 (doi:10.1016/S1360-1385(00)01797-0) 11120476

[78] Meeks J. C. Elhai J. 2002 Regulation of cellular differentiation in filamentous cyanobacteria in free-living and plant-associated symbiotic growth states. Microbiol. Mol. Biol. Rev. 66, 94–121 10.1128/MMBR.66.1.94-121.2002 (doi:10.1128/MMBR.66.1.94-121.2002) 11875129 PMC120779

